# Usefulness of myocardial circunferential strain in acute myocardial infarction for prediction of contractile function recovery: a MRI myocardial tagging study

**DOI:** 10.1186/1532-429X-13-S1-P156

**Published:** 2011-02-02

**Authors:** Esther Pérez David, Loreto Bravo Calero, Maria Jesús Ledesma-Carbayo, José Luis Rubio, Javier Bermejo, Francisco Fernández-Avilés, Javier Lafuente

**Affiliations:** 1 Hospital Gregorio Marañón, Madrid, Spain; 2 ETSIT. Universidad Politécnica de Madrid, Madrid, Spain; 3 ETSIT.Universidad Politécnica de Madrid, Madrid, Spain

## Background

In patients (p) with acute myocardial infarction (AMI), quantitation of myocardial regional function may be useful to differentiate between stunned and necrotic myocardium and to predict ventricular function recovery during follow-up. Different parameters exist to evaluate regional myocardial function such as circunferential /radial strain and displacement. The purpose of this study is to determine the most useful parameter for prediction of contractile function recovery obtained from tagged MRI.

## Methods

12 p admitted to our institution with a first episode of AMI, treated with primary angioplasty and good angiographic result were included in the study (7 anterior and 5 inferior, with mean left ventricular ejection fraction FEVI 44±4%). A cardiac MRI study was performed with a 1.5T unit (Philips Intera), including cine images with left ventricle full coverage, T2-STIR black blood, tagged MRI in basal, mid and apical short axis views, first pass perfusion and 3D late enhancement after administration of 0.2 mmol/kg gadolinium-derived contrast. A second MRI was performed at 6-months' follow-up. Regional ratial/circunferential displacement and strain were quantified with propietary software developed for tagged-MRI postprocessing, based on non-rigid registration algorithms. A 16-segment model was used for analysis. 188 myocardial segments, 80 dysfunctional, were analyzed. Circunferential strain and radial strain were inversely correlated with transmural extension of late enhancement.

In circunferential strain, a cutoff point of 9.3 allowed prediction of ventricular function recovery with sensitivity 83% and specificity 72%.

## Conclusions

Regional myocardial circunferential strain in AMI is strongly correlated with contractile function recovery at 6 months' follow-up.

**Table 1:** Myocardial regional strain versus scar

Late enhancement (% myocardial thickness	Radial strain	Circunferential strain
0-25	11,5 +/-7,0	13,8 +/-4,8
26-50%	12,3+/-4,0	11,8 +/-3,5
51-75%	8,9+/-4,4	11,3 +/-3,7
75-100%	8,2+/-4,6	8,1 +/-5,2

